# Porcine Peripheral Blood Mononuclear Cells (PBMCs): Methods of Isolation, Cryopreservation, and Translational Applications in Human Studies

**DOI:** 10.3390/jcm14103432

**Published:** 2025-05-14

**Authors:** Magdalena Pietrzak, Monika Chaszczewska-Markowska, Magdalena Zemelka-Wiacek

**Affiliations:** 1 Department of Clinical Immunology, Faculty of Medicine, Wroclaw Medical University, 50-367 Wroclaw, Poland; magdalena.zemelka-wiacek@umw.edu.pl; 2 Laboratory of Clinical Immunogenetics and Pharmacogenetics, Hirszfeld Institute of Immunology and Experimental Therapy, Polish Academy of Sciences, 53-114 Wroclaw, Poland; monika.chaszczewska-markowska@hirszfeld.pl

**Keywords:** cryopreservation, isolation, PBMC, peripheral blood mononuclear cells, pig, porcine, swine model

## Abstract

Porcine peripheral blood mononuclear cells (pPBMCs) are increasingly recognized as a valuable model in biomedical and translational research, particularly in contexts directly related to human health and disease. Their immunological features, such as the presence of CD4^+^CD8^+^ double-positive T cells and cytokine expression patterns, exhibit a notable degree of similarity to human immune cells, making them an attractive tool for studying human-relevant immune responses. This review outlines current methodologies for isolating and cryopreserving pPBMCs, with a focus on maintaining high cell viability and functionality. Key technical considerations, including the optimal use of gradient media, appropriate anticoagulants, and standardized freezing/thawing protocols, are discussed in detail. Furthermore, the article highlights the applications of pPBMCs in various research contexts, including vaccine development, inflammation studies, infection models, and xenotransplantation. A comparative perspective is provided to identify similarities and differences between porcine and human PBMCs, supporting the validity of swine as a translational model. Evidence from pPBMC-based studies has shown predictive value for human outcomes, reinforcing their role as a surrogate system for preclinical investigations. Given their anatomical, physiological, and immunogenetic similarities to humans, porcine PBMCs represent a valuable bridge between basic science and clinical application, playing an increasingly important role in translational medicine.

## 1. Introduction

Peripheral blood mononuclear cells (PBMC) are widely used as a representative immune cell population for studying immunological responses and mechanisms in health and disease. They serve as a valuable model for analyzing the body’s response to various challenges, such as pathogens or pharmacological interventions. PBMCs represent a heterogeneous population of peripheral blood cells, primarily including lymphocytes (T helper (Th) CD4+, T cytotoxic (Tc) CD8+, gamma–delta (γδ) T cells, T regulatory (Treg), B, natural killer (NK) cells, monocytes, and dendritic cells (DC) [1,2,3,4,5]. Their main functions involve pathogen recognition, the initiation of immune responses, and the regulation of both innate and adaptive immunity.

Among the various species used in immunological research, the pig has emerged as a particularly valuable model organism. The significance of porcine peripheral blood mononuclear cells (pPBMCs) stems from the unique anatomical, genetic, and immunological similarities between pigs and humans. Swine share comparable immune system components and functional responses, making them more translationally relevant than traditional rodent models in many contexts. This is particularly advantageous in the study of complex human diseases, including inflammatory disorders, infectious diseases, and vaccine responses. Furthermore, the relatively large size of pigs and the ease of repeated blood sampling facilitate longitudinal studies and in-depth immunological analyses. These attributes, combined with the practical benefits of cost-effective husbandry and well-established breeding protocols, make swine and their immune cells highly attractive for biomedical and translational research [6,7]. With the growing interest in this model, there is an increasing need for a thorough understanding of the processes related to the isolation, storage, and application of these cells, paving the way for new discoveries in immunology and translational medicine [8,9].

The use of pPBMCs enables the investigation of species-specific immune processes while offering results that often mirror human immunological responses more accurately than other animal models. This has led to their growing application in vaccine development, xenotransplantation research, and the study of zoonotic infections that pose risks to both swine and human populations. Additionally, pPBMCs provide an essential tool for evaluating the efficacy and safety of novel immunomodulatory therapies before moving to human trials. As such, they represent a critical bridge between basic research and clinical application, supporting the development of next-generation treatments and diagnostic tools [10,11].

The isolation of PBMCs from porcine blood is a process that requires precision to obtain a viable cell population for further analyses. Standard approaches rely on density-based separation techniques, allowing for the extraction of lymphocytes and monocytes from other blood components. This method enables the acquisition of a pure fraction of PBMC, which can then be used in in vitro experiments, such as lymphocyte proliferation assays, surface marker expression analysis, or cytokine production evaluation. A key aspect is the adaptation of protocols to the specific physiology of swine, distinguishing these methods from similar procedures used in other species [12].

Equally important is cryopreservation, a process that allows the long-term storage of cells without compromising their viability or immune responsiveness [13]. The selection of appropriate cryoprotective media plays a crucial role in maintaining cell stability under extreme low-temperature conditions.

The application of pPBMCs in scientific research is extensive and includes studies on innate and adaptive immunity mechanisms, testing new therapeutic strategies, and developing biotechnological products such as vaccines and immunomodulators [14,15,16,17]. Their role in modeling immune responses to viral and bacterial infections, as well as in analyzing reactions to environmental stressors, makes them invaluable across multiple fields. Moreover, pPBMCs are utilized in research on chronic diseases and inflammatory conditions, providing insights into processes with potential implications for human health [15,18,19]. Their availability and suitability for experimental manipulation further enhance their appeal as a research model.

This review aims to compile and systematize current knowledge on pPBMCs, with a particular focus on isolation methods, cryopreservation strategies, and their diverse scientific applications. By discussing these aspects, we aim to highlight both the achievements and gaps in existing approaches, thereby pointing to directions for future research.

## 2. Isolation of pPBMCs

Currently, a standardized protocol for the isolation of pPBMCs has not yet been developed. As a result, methods adapted from human procedures or based on previous comparative studies on porcine cells are commonly used. One of the main challenges during PBMC isolation is sample contamination with granulocytes.

**Figure 1:**
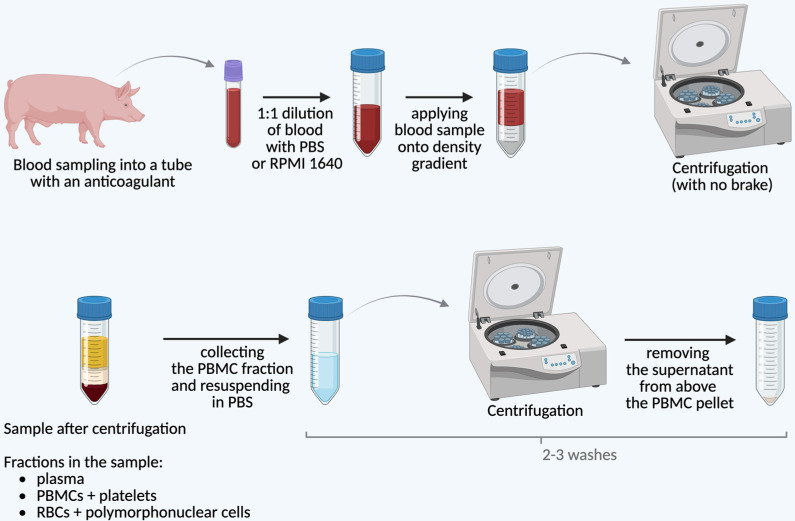
Protocol of PBMC isolation from peripheral blood of swine. Abbreviations: PBMC = peripheral blood mononuclear cells; PBS = phosphate-buffered saline; RBC = red blood cells; RPMI 1640 = Roswell Park Memorial Institute 1640 medium.

Due to the higher density of granulocytes and erythrocytes compared to mononuclear cells, density gradient centrifugation is the most widely used method for separation. A critical step in this approach involves adding an anticoagulant, such as ethylenediaminetetraacetic acid (EDTA), heparin, or sodium citrate, to the blood sample, followed by dilution with an equal volume of phosphate-buffered saline (PBS), which facilitates the effective isolation of PBMCs [20]. Figure 1 provides a comprehensive overview of the PBMC isolation process.

**Figure 2:**
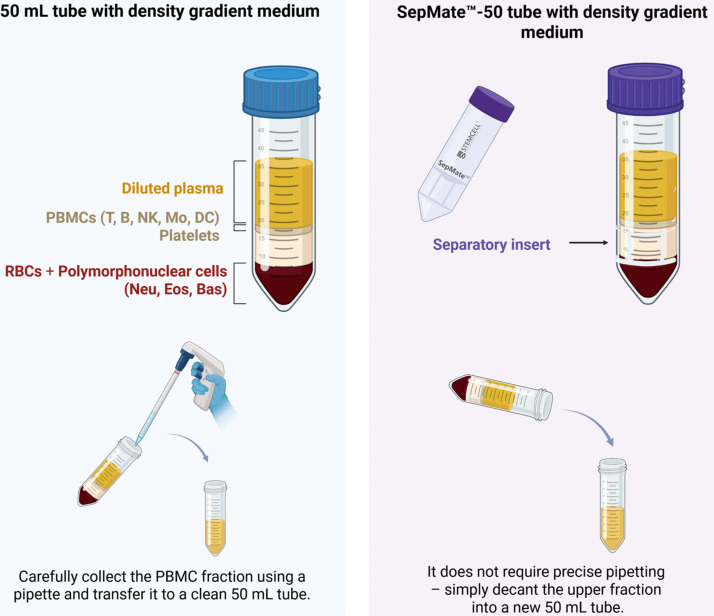
Comparison of blood sample separation using 50 mL tube and SepMate™-50 tube. Abbreviations: B = B lymphocyte; Bas = basophils; DC = dendritic cells; Eos = eosinophils; Mo = monocytes; NK = natural killer cells; Neu = neutrophils; PBMC = peripheral blood mononuclear cells; RBC = red blood cells; T = T lymphocyte.

Various gradient media are available on the market, including Ficoll-Paque™ (Cytiva, GE Healthcare, Marlborough, MA, USA) (density of 1.077 g/mL), Histopaque™ (Sigma-Aldrich, St. Louis, MO, USA) (density of 1.077–1.083 g/mL), Lymphoprep™ (Alere Technologies, Jena, Germany) (density of 1.077 g/mL), or Percoll™ (Cytiva, GE Healthcare, Uppsala, Sweden) (density of 1.070–1.080 g/mL). The choice of the appropriate gradient depends on the swine’s age, as this affects the efficiency of erythrocyte separation. For piglets, a gradient with a density of 1.075 g/mL is used, whereas for adult swine, a density of 1.077 g/mL is recommended [21]. After centrifugation in the density gradient medium, the cells undergo multiple washes in PBS (two or three times), which improves their viability and removes potential contaminants, including platelets [22]. In the case of bovine cells, washing steps help to remove residual platelets that might otherwise interfere with downstream analyses or cellular functions [23]. An alternative to the conventional methods utilizing standard tubes when applying blood onto a density gradient is the use of specialized test tubes equipped with separation inserts. These inserts serve as physical barriers that facilitate the formation of a distinct PBMC layer while minimizing contamination and improving the efficiency of the isolation process. Among these, SepMate™ (Stemcell Technologies, Vancouver, BC, Canada), visible in Figure 2, and Leucosep^®^ (Greiner Bio-One, Kremsmünster, Austria) contain porous inserts that streamline cell separation by preventing the unwanted mixing of layers and reducing the need for precise pipetting, thereby enhancing reproducibility. In contrast, CPT™ (BD Vacutainer^®^, Becton, Dickinson and Company, Franklin Lakes, NJ, USA) offers a fully integrated system with a pre-filled density gradient medium, eliminating the need for additional reagents and manual gradient preparation, making it particularly suited for standardized clinical and high-throughput applications.

A comparison of human cell isolation methods has shown that CPT™ and SepMate™ are effective alternatives to the traditional Ficoll-Paque™ method, providing comparable cell quality and functionality while offering higher cell recovery [24,25]. Moreover, isolated PBMCs using the above tubes exhibit better functionality, particularly in terms of immune responses, increased cytokine production, improved proliferative capacity in response to stimulation, and greater efficiency in cytotoxic assays [24].

## 3. Cryopreservation of pPBMCs

After isolation, PBMCs often require storage for further research or laboratory analyses. Cryopreservation, which involves storing cells at extremely low temperatures, enables their long-term storage and subsequent use in immunological studies, such as analyzing the immune system’s response to infections, vaccines, or therapies. However, to preserve cell viability, phenotype, and functionality, the freezing process must be carried out in a controlled manner, minimizing the risk of damage caused by ice crystal formation or osmotic imbalances [24].

### 3.1. Preparation for Cryopreservation

A key step in preparing cells for freezing is the addition of cryoprotectants. The most commonly used agent is a mixture containing 10% dimethyl sulfoxide (DMSO) and 90% fetal bovine serum (FBS). Alternatively, ready-to-use cryopreservation media, such as PSC (Thermo Fisher Scientific, Waltham, MA, USA) or CryoStor CS10 (Stemcell Technologies, Vancouver, BC, Canada), which also rely on DMSO, are available. Studies indicate that reducing the DMSO concentration to 5% may improve human cell viability after thawing [26]. Furthermore, the comparisons of various cryoprotective media have shown no significant differences in cell survival rates, which remained above 89%. Functional tests, such as interferon gamma (IFN-γ) enzyme-linked immuno-spot ELISpot in response to phytohemagglutinin (PHA) and immunoglobulin G (IgG) ELISpot in response to the mitogenic cocktail resiquimod + interleukin-2 (R848 + IL-2), confirmed that results obtained using cryopreserved pPBMCs are comparable to those obtained using fresh cells [13]. A post-thaw viability of human peripheral blood mononuclear cells (hPBMCs) of 70–80% is considered sufficient for further analyses, minimizing the risk of experimental errors [27].

Cell concentration during freezing is crucial for their survival. A concentration that is too high can lead to mechanical damage caused by ice crystals, while a concentration that is too low reduces the protection provided by cryoprotectants. The recommended concentration is 10^6^–10^7^ PBMCs/mL, although the final choice depends on experimental requirements and the protocol used [28,29,30].

### 3.2. Control of the Freezing Rate

The cooling rate is another critical factor influencing the effectiveness of cryopreservation. Freezing too quickly can lead to osmotic imbalances, while freezing too slowly promotes the formation of large ice crystals, which can damage cells. According to the HIV/AIDS Network Coordination’s standard operating procedure (HANC-SOP) guidelines, the optimal freezing rate is −1 °C/min [31]. In practice, a two-step freezing method is used: in the first step, cells are gradually cooled in ultra-low temperature freezers (ULT) to approximately −80 °C, typically using a controlled-rate freezing container such as Mr. Frosty (manufactured by, e.g., Thermo Fisher Scientific, Waltham, MA, USA, which contains isopropanol to ensure a consistent cooling rate of ~1 °C per minute), and then transferred to the gas phase of liquid nitrogen (LN2), where the temperature drops below −150 °C (HANC-SOP). Storing cells at −80 °C for extended periods can lead to a decline in their viability and functionality, so it is recommended to transfer samples to liquid nitrogen as quickly as possible [32,33,34]. Although pPBMCs are highly sensitive to cryopreservation, long-term storage in liquid nitrogen remains the gold standard [35].

### 3.3. Thawing Process

Thawing is an equally important step in cryopreservation. The rapid thawing of cells in a 37 °C water bath for 2–5 min is recommended. After thawing, it is necessary to remove DMSO, which can be toxic to cells. For this purpose, cells are washed in culture medium by centrifugation at 300× *g* for 5 min and then resuspended in fresh medium [36,37].

## 4. Key Differences Between Human and Porcine PBMCs: Immunological Characteristics and Methodological Considerations

The isolation, characterization, and application of PBMCs differ between humans and swine due to both biological and methodological factors. Understanding these differences is essential for optimizing porcine PBMC (pPBMC) protocols and ensuring accurate data interpretation when comparing results across species.

### 4.1. Immunological and Phenotypic Differences

As of October 2024, the Human Cell Differentiation Molecules (HCDM) organization has assigned designation numbers up to CD371 for the human cluster of differentiation (CD) molecules, reflecting the ongoing advancement in the characterization of the human immune system [38]. In pigs, a landmark study conducted in 2018 identified 359 proteins homologous to human CD markers. Among these, 266 porcine CD molecules have specific antibody sets available, allowing for their detection and functional analysis [39]. Despite this progress, the comprehensive characterization of porcine CD molecules remains an active area of research.

**Figure 3:**
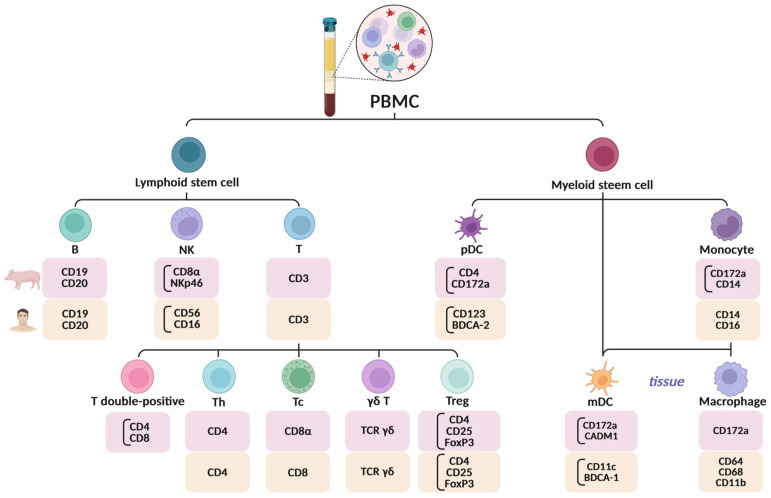
Comparison of the most characteristic markers for porcine and human PBMCs. The bracket indicates that the identification of these cells requires the use of multiple markers, which allows for their accurate differentiation. Abbreviations: B = B lymphocyte; CD = cluster of differentiation; FoxP3 = Forkhead box protein P3; γδ T = gamma–delta T lymphocyte; mDC = myeloid dendritic cell; NK = natural killer cell; pDC = plasmacytoid dendritic cell; PBMC = peripheral blood mononuclear cells; T = T lymphocyte; Tc = cytotoxic T lymphocyte; TCR = T cell receptor; Th = T helper lymphocyte; Treg = regulatory T lymphocyte [39,49,50,51,52,53,54,55,56,57,58].

One of the main differences between pPBMCs and hPBMCs is the presence of a higher number of double-positive T lymphocytes (CD4^+^CD8^+^) (Figure 3) in the peripheral blood of swine, which is not typical for human blood, where T cells are usually distinctly divided into CD4^+^ and CD8^+^ populations [40,41]. These porcine double-positive T cells express CD8αα, as opposed to the CD8αβ found on conventional cytotoxic T cells, and are functionally characterized as a memory T cell population with major histocompatibility complex class II (MHC-II) restriction [40]. A distinct population of circulating double-positive CD4^+^CD8αα^+^ αβ T cells was also identified in pigs. After integrating porcine single-cell RNA sequencing (scRNA-seq) data with human datasets, these porcine double positive T cell clusters showed high prediction scores, aligning with human CD4 central memory T cells (Tcm) [41].

In swine, especially young individuals, a higher number of γδ T cells is observed, whereas in human, Tαβ cells predominate. Specifically, minipigs, particularly juveniles, exhibit a higher proportion of T-cell receptor (TCR)-Tγδ^+^ cells compared to humans, where TCR-Tαβ^+^ cells are more prevalent [42]. NK cells in swine exhibit differences in surface marker expression compared to their human counterparts, which affects their cytotoxic function. In pigs, NK cells show variable expression of NKp46, which helps categorize them into functional subsets, NKp46^−^, NKp46^+^, and NKp46^high^, based on their cytotoxic activity. Additionally, unlike human cells, porcine NK cells express NKp44 at high levels even without activation, with most NK cells expressing it ex vivo. This expression increases further upon stimulation [43,44].

Monocytes in both species share similar properties, though they differ in the expression of certain surface receptors. For example, porcine monocytes express higher levels of CD14 and lower levels of CD16 compared to human monocytes. Furthermore, one study identified two distinct subsets of porcine monocytes based on CD163 expression: CD14^low^/CD163^+^ and CD14^hi^/CD163^−^ [45].

Additionally, responses to cytokines and chemokines may differ, influencing how PBMCs react to infections and inflammatory conditions. A study investigated how pPBMCs respond to stimulation with concanavalin A (Con A) [46]. They observed that the kinetics of cytokine messenger RNA (mRNA) expression differed between whole blood cultures and isolated PBMC cultures. For example, in PBMC cultures, peak mRNA levels occurred at 12 h for IL-2, 24 h for IL-6, 6 h for IL-8, and 12 h for IL-10. In contrast, whole blood cultures exhibited peak mRNA levels for these cytokines at 24 h for IL-2, 12 h for IL-6, 6 h for IL-8, and 24 h for IL-10. Furthermore, PBMC cultures consistently showed higher IL-2 mRNA levels, while whole blood cultures had higher IL-6 mRNA levels [46]. Genetic and evolutionary divergences also lead to the distinct regulation of immunology-related genes, such as those involved in cytokine production. A discovery highlighting species-specific regulatory mechanisms in porcine immune cells was made in a study analyzing how pPBMCs respond to lipopolysaccharide (LPS) and dexamethasone (DEX), revealing species-specific immune regulation. LPS increased the expression of pro-inflammatory cytokines through nuclear factor kappa-light-chain-enhancer of activated B cells (NF-κB) activation while also triggering anti-inflammatory mechanisms. DEX influenced immune responses by both inhibiting and activating different pathways, emphasizing the complex interaction between glucocorticoid signaling and immune regulation [47].

Despite these differences, pPBMCs exhibit many functional similarities to hPBMCs, making them a valuable model in biomedical research. Single-cell transcriptomics studies confirm both homologies and specific differences in gene expression between pPBMCs and hPBMCs. While many immune cell types, including T and B cells, monocytes, and dendritic cells, showed conserved gene expression patterns, notable species-specific differences were identified. In particular, γδ T cells and double-positive CD4^+^CD8^+^ T cells displayed distinct gene expression profiles in pigs [41,48]. The responses to immunological stimulation and immunosuppressive drugs were also analyzed, revealing similar T-cell activation mechanisms in both species. The mechanisms were similar between the two species, but notable differences existed in the activation levels of specific T-cell subsets. Specifically, human CD4^+^ T cells exhibited higher activation levels, whereas minipig CD8^+^ T cells were more abundant and showed greater activation. Additionally, the study found that the effectiveness of immunosuppressive drugs varied depending on the stimuli used, emphasizing the need to consider these differences when evaluating immunomodulatory drugs across species [42]. Understanding these relationships is crucial for fully leveraging the potential of pPBMCs as a model system.

### 4.2. Methodological Considerations in pPBMC Isolation and Handling

Although separation and storage techniques for PBMCs may seem similar across species, the specific characteristics of porcine cells—resulting from differences in blood composition or immune system dynamics—require special attention. The isolation of porcine cells, while generally based on protocols used for hPBMCs, exhibits some differences, which are detailed in Table 1.

Heparin is more commonly used as an anticoagulant for porcine cells, consistent with recommendations for T-cell isolation [59,60]. Minor differences can also be observed in the dilution ratios of collected blood. Human blood is typically diluted 1:1 with PBS or Hank’s balanced salt solution (HBSS), while porcine blood is diluted 1:1 or 1:2, most often using PBS or RPMI 1640.

Regardless of the density gradient used, variability in centrifugation parameters is observed in protocols for porcine cells. Higher centrifugal acceleration (*g*) values are often used compared to standard procedures for hPBMCs. These differences likely arise from the distinct biological properties of the cells. The recommended temperature for both cell types is 20–25 °C, and the centrifugation time is typically around 30 min, which is commonly accepted for both swine and human.

The number of washes depends mainly on the degree of platelet contamination and the turbidity of the supernatant. For pPBMCs, 2–3 washes are typically performed, though centrifugation parameters can be difficult to standardize due to noticeable differences or a lack of precise guidelines in the literature.

**Table 1:** The isolation of PBMC from swine and human blood: a comparative analysis. Abbreviations: CD = citrate dextrose; EDTA = ethylenediaminetetraacetic acid; HBSS = Hank’s balanced salt solution; n.p. = not provided; PBS = phosphate-buffered saline; PLT = platelets; RPMI 1640 = Roswell Park Memorial Institute 1640 medium.

Species	Ref.	Anti- Coagulant	Ratio and Diluent	Density Gradient	Cell Separation Parameters	Number of Washes and Washing Solution	Washing Parameters
swine	[61]	EDTA	1:2 PBS	Histopaque^®^-1077	400× *g* 30 min; RT	2 washes, PBS	250× *g* 10 min; RT
	[62]	heparin	1:1 RPMI 1640	Histopaque^®^-1077	1100× *g* 25 min; RT	2 washes, n.p.	n.p.
	[63]	heparin	n.p.	BD Vacutainer^®^ CPT™	500× *g* 30 min	3 washes, PBS	n.p.
	[64]	heparin	1:2 RPMI 1640	Histopaque^®^-1077	1100× *g* 25 min	2 washes, RPMI 1640	n.p.
	[65]	n.p.	1:1 PBS	Histopaque^®^-1077	2200 rpm RT	3 washes, PBS	2500 rpm 15 min; 4 °C
	[66]	heparin	n.p.	BD Vacutainer^®^ CPT™	1500× *g* 30 min; RT	2 washes, PBS	700× *g* 10 min
	[67]	EDTA	1:1 PBS + 20% CD + 2% FBS	SepMate™-50 tube with Lymphoprep™	1100× *g* 10 min	until PLT removal, PBS + 20% ACD + 2% FBS	n.p.
	[68]	EDTA	1:1 PBS	Ficoll-Paque™	400× *g* 30 min; 25 °C	3 washes, n.p.	1. 300× *g* 10 min; 4 °C; 2. 250× *g* 10 min; 4 °C
	[69]	heparin	1:1 HBSS	Ficoll-Hypaque	472× *g* 30 min; RT	1 wash, HBSS	n.p.
	[70]	heparin	2:1 PBS	Ficoll-Paque™ PLUS	1455× *g* 30 min; RT	3 washes, PBS	930× *g* 5 min; 4 °C
human	[71]	n.p.	1:1 HBSS	Ficoll-Hypaque	2000 r/min 20 min	2 washes, HBSS	n.p.
	[72]	EDTA	1:1 PBS	Ficoll-Paque™ PLUS	400× *g* 30 min; 20 °C	1 wash, PBS	300× *g* 10 min; 20 °C
	[73]	EDTA	1:1 PBS	LSM™	400× *g* 30 min	2 washes, PBS	n.p.
	[74]	heparin	n.p.	Histopaque^®^-1077	2000 r/min 20 min; 4 °C	2 washes, PBS	1500 r/min 10 min; 4 °C
	[75]	n.p.	1:1 PBS + 2% FBS	Lymphoprep™	800× *g* 20 min; RT	n.p.	n.p.
	[76]	n.p.	PBS	Lympholyte^®^	1500 r/min 30 min; RT	n.p. PBS	n.p.
	[77]	EDTA	1:1 PBS	Histopaque^®^-1077	800× *g* 30 min; 22 °C	1 wash, PBS	300× *g* 3 min; 22 °C
	[78]	EDTA	1:1 PBS	Ficoll-Paque™ PLUS	400× *g* 30 min; 25 °C	2 washes, PBS	300× *g* 10 min; 25 °C
	[79]	EDTA	1:1 PBS + 2% FBS	Lymphoprep™	800× *g* 20 min; RT	until the supernatant is clear, PBS	n.p.
	[80]	sodium citrate	1:1 PBS	Lymphoprep™	1. 160× *g* 20 min; RT; 2. 800× *g* 20 min	3 washes, RPMI	n.p.

## 5. Utilization of Porcine PBMCs

PBMCs are increasingly being used as a translational model in biomedical research, including preclinical safety assessments and drug development. This is due to their significant physiological, anatomical, and genetic similarities to human [42,81,82,83]. Swine are characterized by their organ sizes, metabolic processes, and functions of the cardiovascular, digestive, and immune systems that are comparable to those of humans, making them a more suitable model than smaller laboratory animals, such as rodents [82,84,85]. Additionally, the porcine genome shows greater similarity to the human genome, facilitating disease modeling and the testing of gene therapies. Swine are also widely used in toxicological, pharmacokinetic, and surgical studies, as well as in the testing of medical implants [81,85,86,87,88]. Despite challenges such as higher maintenance costs and ethical considerations, swine remain a valuable tool in biomedical research, contributing to the development of effective therapies for humans.

### 5.1. Immunological and Translational Applications of pPBMCs

Porcine PBMCs serve as a valuable model for dissecting fundamental immune mechanisms and testing therapeutic interventions, making them ideal for translational immunology research. These cells are widely used to study immune responses to various stimuli, providing insights into the mechanisms of innate and adaptive immunity. For instance, stimulation with LPS, a component of Gram-negative bacterial cell walls, induces the production of pro-inflammatory cytokines such as tumor necrosis factor alpha (TNF-α), IL-1β, and IL-6 through activation of the toll-like receptor 4 (TLR4) pathway, closely mirroring responses in humans [15,89,90]. Similarly, polyinosinic:polycytidylic acid (Poly I:C), a viral double-stranded RNA mimic, activates the TLR3 pathway, leading to interferon production and antiviral gene expression [15].

Transcriptomic analyses using RNA sequencing (RNA-seq) have identified 603 genes specific to LPS stimulation, 254 to Poly I:C, and 882 to exposure to various microorganisms (EM), with 38 genes common across all three stimuli. Functional analyses show that LPS and Poly I:C activate pathways involved in inflammation and immune signaling, while EM exposure enhances metabolic responses, suggesting relevance for modeling sepsis, endotoxemia, and viral infections [15].

In vaccine development, pPBMCs play a crucial role in assessing immunological efficacy. Post-vaccination, the production of antigen-specific antibodies, T-cell proliferation, and cytokine secretion are analyzed [82,91,92,93,94,95,96,97]. Similarly, studies on influenza vaccines utilize PBMCs to assess cellular responses, which may contribute to the development of universal vaccines. These studies underscore the importance of PBMCs in optimizing vaccine formulations and understanding the mechanisms of immune memory.

Porcine PBMCs are also used to study the effects of immunomodulatory factors, which can enhance or suppress the immune response. For example, studies on enrofloxacin have shown that this antibiotic can transiently inhibit the production of pro-inflammatory cytokines in LPS-stimulated cells, suggesting immunomodulatory effects beyond its antibacterial properties [89]. Similarly, corticosteroids, commonly used to treat inflammatory conditions, inhibit cytokine production in PBMCs, providing a model for human therapy research [47,98].

### 5.2. Pathogen-Specific Infectious Disease Research Using pPBMCs

Porcine PBMCs are extensively used to investigate host–pathogen interactions in the context of viral and bacterial infections, especially those with zoonotic potential or significant impact on swine health. One of the most studied pathogens is the porcine reproductive and respiratory syndrome virus (PRRSV), which causes immunosuppression and chronic infection by modulating cytokine production and disrupting T-cell function [94,96,97].

Studies on vaccines against porcine reproductive and respiratory syndrome virus (PRRSV) have shown that the production of IFN-γ by virus-specific T cells correlates with protection against infection. A particularly important role is played by cytotoxic CD4^+^ T cells, which, upon in vitro restimulation, exhibit the increased expression of activation markers such as CD107a and IFN-γ, suggesting their critical role in protection against PRRSV-1 infection [99].

Another significant pathogen is the African swine fever virus (ASFV), characterized by high mortality rates. ASFV induces the production of pro-inflammatory cytokines and chemokines in pPBMCs, which correlates with disease severity [100]. Additionally, research on other viruses, such as classical swine fever virus (CSFV) and porcine circovirus type 2 (PCV2), utilizes pPBMCs to understand host–pathogen interactions and the kinetics of immune responses [101,102,103,104].

Porcine PBMCs are also a valuable tool in research on bacterial diseases, helping to elucidate the complex interactions between the host and pathogen. An example is *Streptococcus suis*, a zoonotic pathogen that causes meningitis in both swine and human. Infection with this pathogen induces both pro-inflammatory and anti-inflammatory cytokines in pPBMCs, with IL-10 playing a key role in modulating the inflammatory response [105,106]. Another important pathogen is *Salmonella*, particularly *Salmonella typhimurium*, which activates macrophages and dendritic cells in pPBMCs, initiating the host immune response [89]. These studies provide insights into the body’s defense mechanisms and potential therapeutic strategies.

### 5.3. Xenotransplantation

Transplantation, as a field of medicine focused on organ and tissue grafting, has long faced challenges such as donor shortages and graft rejection by the recipient’s immune system. In response to these challenges, scientists are increasingly developing xenotransplantation, where swine are considered promising donors due to the anatomical and physiological similarities of their organs—such as the heart and kidneys—to those of humans. However, the key obstacle remains the immunological barrier: human immune cells quickly recognize swine tissues as foreign, leading to rejection. In this context, pPBMCs play an indispensable role in research on immunological mechanisms, testing therapies, and enhancing cross-species compatibility.

Mixed porcine lymphocyte cultures with human cells are useful for assessing proliferative responses, indicating the potential risk of graft rejection [107,108,109]. The primary immunological barrier is the galactose-α-1,3-galactose (α-Gal) antigen, produced by the enzyme α-1,3-galactosyltransferase (GGTA1), which triggers natural antibody responses in humans. Genetic modifications, such as knockout of the GGTA1 gene, reduce α-Gal expression, thereby decreasing immunological reactivity [107,109,110,111,112,113,114,115,116].

Further genetic modifications, including the introduction of human complement regulatory proteins such as CD55 and CD59, are being tested in pig cells to enhance their resistance to complement-mediated lysis when exposed to human immune components. These modifications aim to address one of the major immunological barriers in xenotransplantation—hyperacute rejection—by inhibiting the activation and amplification of the complement cascade. Using hPBMCs as an in vitro model, researchers are evaluating the protective effects of these transgenes on porcine endothelial and other relevant cell types. The expression of CD55 interferes with the formation of C3/C5 convertases, while CD59 inhibits the formation of the membrane attack complex (MAC), both crucial mechanisms in mitigating complement-induced cytotoxicity. These approaches are being explored in combination with other genetic alterations, such as the deletion of α1,3-galactosyltransferase (GGTA1), to produce multi-transgenic pigs that are more compatible with the human immune system and potentially suitable for clinical xenotransplantation [107,109]. Additionally, PBMCs help elucidate the role of other antigen-presenting cells, such as porcine dendritic cells (DC research indicates that inflammatory signals, such as TNF-α, accelerate porcine DC maturation, enhancing their ability to activate human T cells and increasing the risk of graft rejection). This highlights the importance of controlling inflammation to reduce immune activation [117]. Conversely, transforming growth factor β1 (TGF-β1) has been shown to influence the differentiation of porcine monocytes into Langerhans cell-like dendritic cells (MoLCs), a specialized subset of antigen-presenting cells. These porcine MoLCs exhibit distinct surface markers and functional properties, including high expression of major histocompatibility complex (MHC) molecules and co-stimulatory proteins, which enhance their capacity to present antigens and activate T cells. Their potent immunostimulatory function positions them as key contributors to immune surveillance and rejection processes in xenotransplantation [109]. These advancements, tested using pPBMCs, contribute to reducing the risk of graft rejection.

pPBMCs are a valuable in vitro model for evaluating the efficacy of immunosuppressive drugs in xenotransplantation research. One such compound, rapamycin, has been shown to significantly inhibit pPBMC proliferation in response to mitogenic stimuli, such as concanavalin A (ConA) or phytohemagglutinin (PHA), by interfering with the mechanistic target of rapamycin (mTOR) signaling pathway. This suppression helps mitigate immune activation that can lead to graft rejection. Notably, studies examining pPBMCs restimulated with porcine circovirus type 2 (PCV2) antigen—a model for antigen-specific immune responses—demonstrated that rapamycin effectively reduces antigen-driven lymphocyte proliferation as well. These findings emphasize that the efficacy of immunosuppressive agents like rapamycin is not uniform but highly dependent on the nature of the immune challenge. As a result, tailoring immunosuppressive therapy to match the specific immunological context—whether it involves alloantigen, xenoantigen, or viral antigen stimulation—is crucial for optimizing graft acceptance while minimizing immune suppression-related complications [42].

Strategies for inducing tolerance, such as hematopoietic stem cell transplantation or the expression of immune-inhibitory molecules, are being intensively explored using pPBMC models [118,119,120]. Monitoring immune responses post-transplantation using pPBMCs allows for the identification of early signs of rejection or tolerance, which is crucial for clinical applications [97,110,112,121].

## 6. Conclusions

Porcine PBMCs have emerged as a critical tool in modern biomedical research, offering a robust, reproducible, and physiologically relevant model for studying immune function, disease mechanisms, and therapeutic responses. The isolation and preservation techniques described herein ensure high cell viability and functional integrity, allowing for a broad range of experimental applications. The unique immunophenotypic characteristics of pPBMCs, including the presence of CD4^+^CD8^+^ T cells and species-specific cytokine expression patterns, provide both opportunities and challenges when extrapolating findings to human systems. However, comparative studies using advanced transcriptomic and functional analyses have revealed a considerable overlap in immune responses between porcine and human PBMCs. This reinforces the utility of swine as a translational model, particularly in fields such as vaccine development, immunomodulatory therapy, infectious disease research, and xenotransplantation. Advances in in vitro systems utilizing pPBMCs have significantly expanded their potential for use in preclinical studies, particularly where human samples are limited or ethically restricted.

As the demand for reliable large-animal models grows, the porcine immune system—especially through pPBMC-based approaches—offers a practical and physiologically relevant platform for bridging basic research with clinical applications. Thanks to their anatomical, physiological, and immunogenetic similarities to humans, porcine PBMCs are becoming an increasingly important tool in translational medicine.

## Abbreviations

The following abbreviations are used in this manuscript:

α-Gal	alpha-galactosidase
γδ T	gamma–delta T cell
ASFV	African Swine Fever Virus
B	B lymphocyte
CD	cluster of differentiation
DC	dendritic cell
DEX	dexamethasone
DMSO	dimethyl sulfoxide
EDTA	ethylenediaminetetraacetic acid
ELISpot	enzyme-linked ImmunoSpot
FBS	fetal bovine serum
GGTA1	gene encoding alpha1,3-galactosyltransferase
HANC-SOP	HIV/AIDS Network Coordination standard operating procedures
HBSS	Hank’s balanced salt solution
hPBMC	human peripheral blood mononuclear cell
IFN-γ	interferon gamma
IL	interleukin
LN2	liquid nitrogen
LPS	lipopolysaccharide
mRNA	messenger RNA
MHC	major histocompatibility complex class
NK	natural killer cell
NKp	natural killer protein
n.p.	not provided
PBMC	peripheral blood mononuclear cell
PBS	phosphate-buffered saline
PCV2	porcine circovirus type 2
PolyI:C	polyinosinic:polycytidylic acid
pPBMC	porcine peripheral blood mononuclear cell
PRRSV	porcine reproductive and respiratory syndrome virus
RBC	red blood cell
RPMI 1640	Roswell Park Memorial Institute 1640 (cell culture medium)
RT	room temperature
Tc	cytotoxic T cell
Th	T helper cell
TLR	toll-like receptor
TNF-α	tumor necrosis factor alpha
Treg	regulatory T cells
